# Extraction Optimization, Characterization, and Bioactivities of Polysaccharides from *Pinelliae Rhizoma Praeparatum Cum Alumine* Employing Ultrasound-Assisted Extraction

**DOI:** 10.3390/molecules22060965

**Published:** 2017-06-09

**Authors:** Yu-Jie Liu, Xue-Lin Mo, Xiao-Zhang Tang, Jiang-Hua Li, Mei-Bian Hu, Dan Yan, Wei Peng, Chun-Jie Wu

**Affiliations:** 1 College of Pharmacy, Chengdu University of Traditional Chinese Medicine, Chengdu 611137, China; liu-1567@163.com (Y.-J.L.); moxuelinde@126.com (X.-L.M.); tangxiaozhang333@163.com (X.-Z.T.); lijianghua1413@126.com (J.-H.L.); hmbcdtcm@163.com (M.-B.H.); yan910459@163.com (D.Y.); pengwei002@126.com (W.P.); 2 Key Research Laboratory of Traditional Chinese Medicine Processing Technology, State Administration of Traditional Chinese Medicine of People’s Republic of China, Chengdu 611137, China

**Keywords:** polysaccharides, *Pinelliae Rhizoma Praeparatum Cum Alumine*, ultrasound-assisted extraction, response surface methodology, bioactivities

## Abstract

In this study, the ultrasound-assisted extraction of polysaccharides (PSA) from *Pinelliae Rhizoma Praeparatum Cum Alumine* (PRPCA) was optimized by response surface methodology (RSM). The structural characteristics of PSA were analyzed by UV-vis spectroscopy, infrared spectroscopy, scanning electron microscopy, high performance gel permeation chromatography and high performance liquid chromatography, respectively. In addition, antioxidant and antimicrobial activities of PSA were studied by different in vitro assays. Results indicated that the optimal extraction conditions were as follows: the ratio of water to raw of 30 mL/g, extraction time of 46.50 min, ultrasonic temperature of 72.00 °C, and ultrasonic power of 230 W. Under these conditions, the obtained PSA yield (13.21 ± 0.37%) was closely agreed with the predicted yield by the model. The average molecular weights of the PSA were estimated to be 5.34 × 10^3^ and 6.27 × 10^5^ Da. Monosaccharide composition analysis indicated that PSA consisted of mannose, galactose uronic acid, glucose, galactose, arabinose with a molar ratio of 1.83:0.55:75.75:1.94:0.45. Furthermore, PSA exhibited moderate antioxidant and antibacterial activities in vitro. Collectively, this study provides a promising strategy to obtain bioactive polysaccharides from processed products of herbal medicines.

## 1. Introduction

*Pinellia ternata* (Thunb.) Breit (PTB) (Araceae) is a famous traditional Chinese medicinal plant which is mainly distributed in the Sichuan, Guizhou and Anhui provinces of China [1,2]. The dried tuber of PTB, named Banxia (BX) in Chinese, has been used as a traditional Chinese Medicine (TCM) for more than 2000 years for relieving cough and inflammation [3]. Investigations have proved that BX possesses multiple pharmacological activities, including antitumor, antioxidant, antitussive, expectorant, antiemetic, antibacterial and anti-inflammatory effects, etc. [4,5]. Previous studies have revealed BX contains abundant chemical constituents, such as alkaloids, lectins, fatty acids, cerebrosides, volatile oils, flavonoids, sterols, and phenylpropanoids [6]. *Pinelliae Rhizoma Praeparatum Cum Alumine* (PRPCA) is a traditional processed product of BX (raw BX processed with alumen as adjuvant material) mainly used for treating phlegm [7,8]. However, limited studies regarding the chemical constituents and pharmacological activities of PRPCA have been performed in the literature.

Recently, increasing polysaccharides with various pharmacological activities have been found in natural plants [9,10]. It’s reported that these effects are mainly due to the antioxidant activity of polysaccharides, and some antioxidant polysaccharides have been used for the treatment of diabetes, cancer, and Alzheimer’s diseases [11]. Previous studies have also reported that polysaccharides from natural plants demonstrate strong antimicrobial activity against different pathogens, which is worthy of further research [12,13]. In addition, polysaccharides from BX and their anti-tumor activity have been reported in previous researches [4,14]. However, to the best of our knowledge, no study has investigated the polysaccharides from PRPCA and their bioactivities.

In this study, ultrasound-assisted extraction (UAE) was employed and the extraction conditions were optimized by using response surface methodology (RSM) to obtain higher extraction yields of PSA with more effective bioactivities from PRPCA. Then Fourier transform-infrared spectroscopy (FT-IR), scanning electron microscopy (SEM), molecular weight determination and monosaccharide composition analysis were carried out to study the structural characteristics of the obtained PSA. Additionally, the antioxidant and antibacterial activities were performed in vitro to investigate the bioactivities of PSA.

## 2. Results and Discussion

Ultrasound-assisted extraction (UAE) has been developed as a novel technique with low cost, low temperature and maximum extraction yield in the extraction of polysaccharides from different plant materials [15]. The main mechanism of UAE is based on cavitation phenomena and a mechanical mixing effect. Ultrasonic energy can cause a disruption of plant cell walls owing to the collapse of cavitation bubbles at the surface of the solid matrix. Then mass transfer is enhanced, allowing greater penetration of the solvent into the solid matrix, and increasing the contact surface area between the solid and liquid phase [16,17].

Compared with conventional extraction, UAE has advantages of simplifying manipulation, reducing processing time and solvent volume usage, extraction at lower temperatures, higher purity and greater yields of the final product, and faster extraction rates, etc. [18,19]. However, the extraction yield of polysaccharides by UAE is often influenced by parameters including ultrasonic power, ultrasonic temperature, extraction time and ratio of water to raw material [11,20]. Therefore, it is important to optimize the extraction conditions to obtain higher yield and reduce degradation of polysaccharides. Response surface methodology (RSM) has been used as an effective statistical methods for optimization of complicated processes and widely employed in polysaccharides extraction process because of less laborious and time-consuming than other methods [21,22]. In this study, a BBD with three factors and three levels was employed for the optimization of ultrasound-assisted extraction (UAE) of PSA. The results indicated that UAE and RSM are feasible and reliable methods for the extraction of PSA.

### 2.1. Single-Factor Experiment Analysis

#### 2.1.1. Effect of Ultrasonic Power on PSA Yield

**Figure 1:**
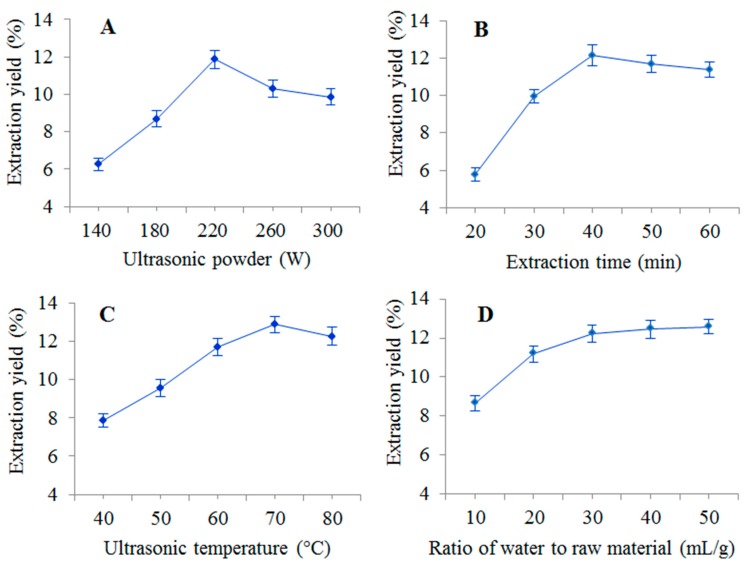
Effects of different extraction parameters (**A**: ultrasonic power; **B**: extraction time; **C**: ultrasonic temperature; **D**: ratio of water to raw material) on the yield of PSA.

Ultrasonic power is reported to be an important extraction parameter that significantly affects the extraction yield [15]. The effect of ultrasonic power on PSA yield is shown in Figure 1A. In this study, ultrasonic powers of 140, 180, 220, 260, and 300 W were investigated, while other parameters (extraction time 40 min, ultrasonic temperature 60 °C, and ratio of water to raw material 30 mg/L) were held constant. It was found that the PSA yield increased linearly and reached a maximum value at 220 W, then decreased as the ultrasonic power further increased up to 300 W. As a result, ultrasonic power of 180–260 W was selected for further experiments.

#### 2.1.2. Effect of Extraction Time on PSA Yield

The effect of different extraction times of 20, 30, 40, 50 and 60 min on the yield of PSA were studied with the other factors kept constant at the central points, and the results are shown in Figure 1B. The yield of PSA increased sharply with the increase of extraction time, and reached a maximum value at 40 min. Then the PSA yield slightly decreased when the extraction time further raised to 60 min. This finding showed that yield of polysaccharides started to maintain a dynamic equilibrium with increasing extraction time, and excessively lengthening the ultrasonic irradiation time will cause degradation of PSA [11,23]. Thus, an extraction time of 30–50 min was used in the RSM experiments.

#### 2.1.3. Effect of Ultrasonic Temperature on PSA Yield

As shown in Figure 1C, the ultrasonic temperature (40, 50, 60, 70 and 80 °C) was varied while the other parameters (ultrasonic power 220 W, extraction time 40 min and ratio of water to raw material 30 mL/g) were kept constant. The extraction yield of PSA increased rapidly (40 to 70 °C), reached a maximum amount at 70 °C and decreased thereafter. This phenomenon indicates that polysaccharide solubility increases with the temperature, but a higher temperature can induce thermal instability and degradation [10]. Therefore, an extraction temperature range of 60–80 °C was considered to be optimal for further experiments.

#### 2.1.4. Effect of Ratio of Water to Raw Material on PSA Yield

Extraction parameters were fixed at ultrasonic power of 220 W, extraction time of 40 min and extraction temperature of 60 °C, while the ratio of water to raw material was studied at 10, 20, 30, 40, and 50 mL/g. As shown in Figure 1D, the yield of PSA increased with the increase of ratio of water to raw material from 10 to 30:1. However, when the ratio of water to raw material continued to increase, the yield of PSA did not change much. This would result in solvent and time waste when the ratio of water to raw material was too high [24], hence, a ratio of water to raw material of 30 mL/g was selected as optimum value for the RSM experiments.

### 2.2. Optimization of Extraction Conditions

#### 2.2.1. Model Fitting Analysis

Based on results of preliminary single-factor experiments above, three factors including extraction time (*X*_1_), ultrasonic temperature (*X*_2_) and ultrasonic power (*X*_3_) were selected for response surface methodology (the ratio of water to raw material was fixed at 30 mL/g). A three-factor Box-Behnken design (BBD) were empolyed for optimal extraction conditions. As shown in Table 1, a total of 17 experiments were performed for different combinations of the extraction factors using BBD by Design Expert software of version 8.0.6.1 (Stat-Ease Inc., Minneapolis, MN, USA). In these experiments, five runs (3, 6, 7, 15 and 16) with the factors set at central levels were used for evaluating the stability of the experiment, and the other 12 runs were performed for analysis. The yield of PSA was ranged from 6.13% to 12.88%, and reached the maximum value at extraction time of 40 min, ultrasonic temperature of 70 °C and ultrasonic power of 220 W. The predicted response (PSA yield) could be obtained by the following second-order polynomial Equation (1):

Yield = 14.61 + 1.53 *X*_1_ + 1.24 *X*_2_ + 1.14 *X*_3_ − 0.79 *X*_1_*X*_2_ − 0.83 *X*_1_*X*_3_ + 0.10 *X*_2_*X*_3_ − 0.93 *X*_1_^2^ − 1.85 *X*_2_^2^ − 1.42 *X*_3_^2^ (1)

**Figure 2:**
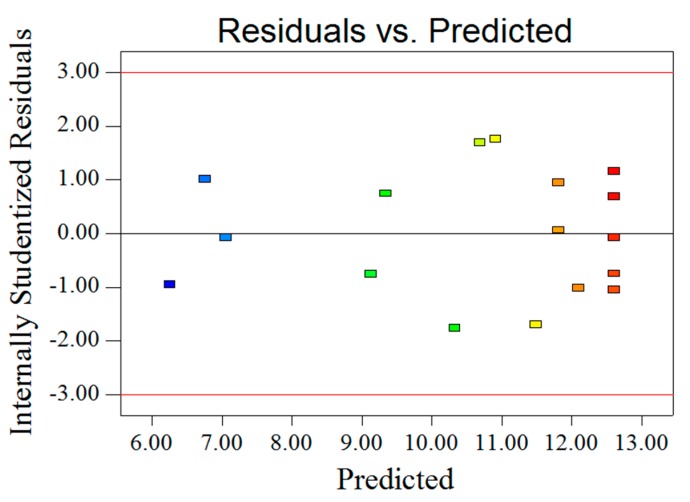
Model adequacy plots.

Analysis of variance (ANOVA) was applied to evaluate the significance of the obtained experimental data and to analyze the adequacy and the fitness of the model [25,26]. According to the results in Table 2, the high *F*-value (121.22) and low *p*-value (<0.0001) suggested that the regression models are very significant. The lack of fit was not significant (*F*-value = 2.03, *p*-value = 0.2523 > 0.05), indicating that the model is adequate for predicting the yield of PSA (Figure 2). The high coefficient (*R*^2^ = 0.9936) and high adjusted determination coefficient (Adj *R*^2^ = 0.9854) indicating a high correlation between the predicted and experimental values [27]. A low coefficient variation (C.V.) (2.09) and a high Adeq precision (31.341) showed a very high degree of precision and good reliability of the experimental values. Furthermore, the *p*-values were used to check the significance of each coefficient, the larger *F*-value and smaller *p*-value means the more significant of coefficient [28]. As a result, the linear coefficients (*X*_1_, *X*_2_ and *X*_3_), interaction coefficients (*X*_1_*X*_2_ and *X*_1_*X*_3_) and quadratic coefficients (*X*_1_^2^, *X*_2_^2^, and *X*_3_^2^) were significant (*p* < 0.05), whereas the other coefficient (*X*_2_*X*_3_) were insignificant (*p* > 0.05).

#### 2.2.2. Response Surface Analysis of Extraction Yield of PSA

**Figure 3:**
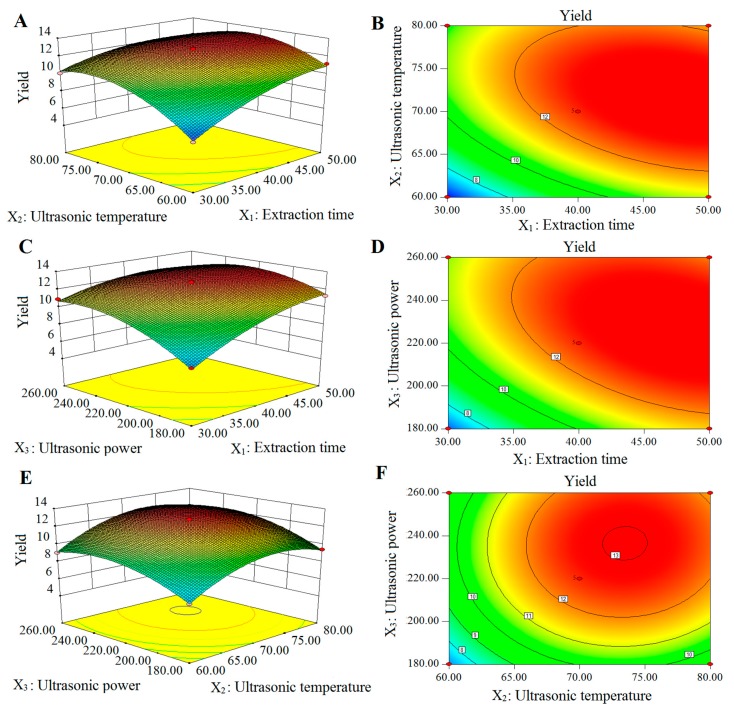
The 3D response surface and 2D contour plots showing the effects of extraction factors on the yield of PSA. (**A**,**B**) were 3D response surface and 2D contour plots showing the effects of extraction time and ultrasonic temperature on the yield of PSA; (**C**,**D**) were 3D response surface and 2D contour plots showing the effects of extraction time and ultrasonic power on the yield of PSA; (**E**,**F**) were 3D response surface and 2D contour plots showing the effects of ultrasonic temperature and ultrasonic power on the yield of PSA.

Three-dimensional (3D) response surface and two-dimensional (2D) contour plots were provided as graphical representations of the regression equation (Figure 3). The interaction effects between the variables were exhibited by the shapes of the contour plots. An elliptical contour plot indicates a significant interaction between corresponding variables, whereas a circular contour plot indicates the interaction between the corresponding variables was insignificant [29,30]. The effects of extraction time, ultrasonic temperature and their interaction on the yield of PSA are shown in Figure 3A,B. The PSA yield increased when the extraction time increased in the range of 30–47.55 min and the ultrasonic temperature increased in the range of 60–71.83 °C. Then the yield decreased after 47.55 min and 71.83 °C.

The contour plot was elliptical, indicating the interaction between extraction time and ultrasonic temperature was significant. The effects of extraction time and ultrasonic power on yield of PSA are shown in Figure 3C,D. A maximum PSA yield was obtained at the extraction time of approximately 47.40 min and ultrasonic power of approximately 227.50 W. The elliptical shape of the contour plot showed that the interactions of the two variables were significant. As shown in Figure 3E,F, the effect of ultrasonic power and ultrasonic temperature on the yield of PSA were evaluated at fixed extraction time of 40 min. A maximum yield (approximately 13.05%) of PSA was obtained when the ultrasonic temperature was set at approximately 73.45 and ultrasonic power at approximately 236.44 W. However, the contour plot was circular, indicating that the mutual interaction between ultrasonic power and ultrasonic temperature was insignificant.

#### 2.2.3. Optimization and Verification of Extraction Conditions

The optimum extraction conditions for maximum extraction yield of PSA proposed by the Design-Expert software were as follows: extraction time of 46.38 min, ultrasonic temperature of 72.04 °C, and ultrasonic power of 228.81 W. Validation experiments (*n* = 3) were carried out under these conditions with slight modifications: extraction time of 46.50 min, ultrasonic temperature of 72.00 °C, and ultrasonic power of 230 W. Under these conditions, the actual yield of PSA obtained was 13.21 ± 0.37%, which highly matched the yield (13.35%) predicted by the regression model. The results indicated that the model was accurate and adequate in predicting PSA extraction conditions.

### 2.3. Results of UV and IR Analysis

**Figure 4:**
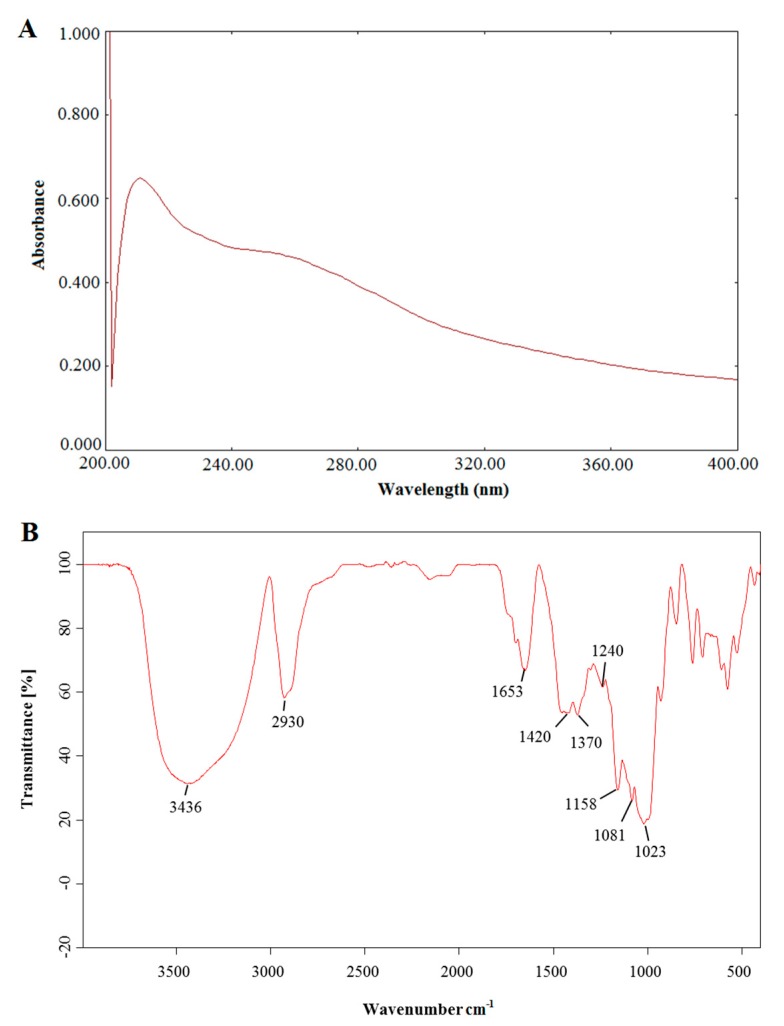
UV-vis spectra (**A**) and FT-IR spectra (**B**) of PSA.

The UV results are shown in Figure 4A. There was a weak absorption peak at 260–280 nm, indicating that the PSA sample contained polypeptides or proteins [31]. FT-IR was applied to analyze the PSA and the results are shown in Figure 4B. The band around 3436 cm^−1^ represented the stretching vibration of the hydroxyl groups in the constituent sugar residues [32]. The band at approximately 2930 cm^−1^ represented the C-H asymmetric stretching vibration in the sugar ring [33]. The band at approximately 1653 cm^−1^ was due to C-O asymmetric stretching vibration [34]. The bands at 1420, 1370 and 1240 cm^−1^ were due to C-O stretching vibrations and O-H deformation vibrations [35]. Furthermore, the strong absorption band between 1200 and 1000 cm^−1^ (1158, 1081 and 1023 cm^−1^) were assigned to the stretching vibration of C-O-C and C-O-H bonds [36].

### 2.4. SEM Analysis

**Figure 5:**
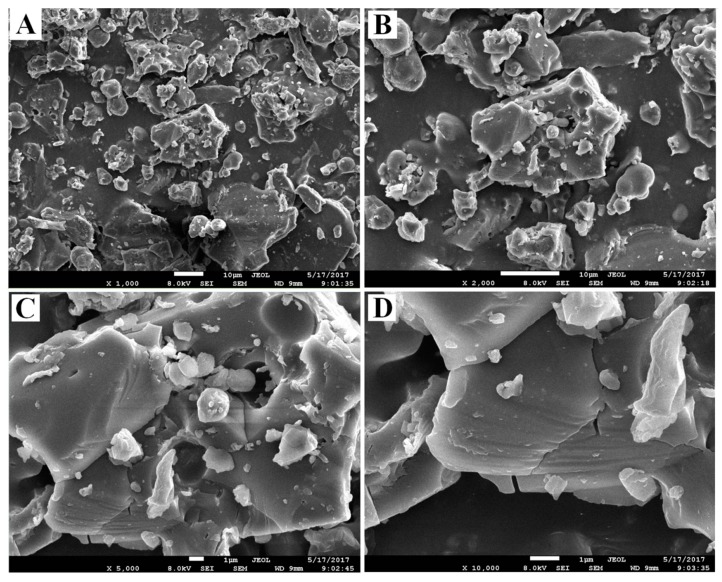
SEM images of PSA. (**A**) Morphology at 1000× (scalebar is 10 μm); (**B**) Morphology at 2000× (scalebar is 10 μm); (**C**) Morphology at 5000× (scalebar is 1 μm); (**D**) Morphology at 10,000× (scalebar is 1 μm).

The microstructure of the PSA was investigated by SEM at different magnifications (1000×, 2000×, 5000× and 10,000× (Figure 5). It has been reported that different surface topography of the polysaccharides can be influenced by different physicochemical properties and types of extraction method [9]. In this study, PSA was extracted from PRPCA by ultrasound-assisted extraction. As shown in Figure 4, PSA showed a fragmented and irregularly shaped morphology at magnifications of 1000×, 2000×. The surface of PSA when observed at magnifications of 5000× and 10,000× appeared to be relatively smooth, with some honeycombed cavities and fine stripes.

### 2.5. Molecular Weight and Monosaccharides Composition of PSA

**Figure 6:**
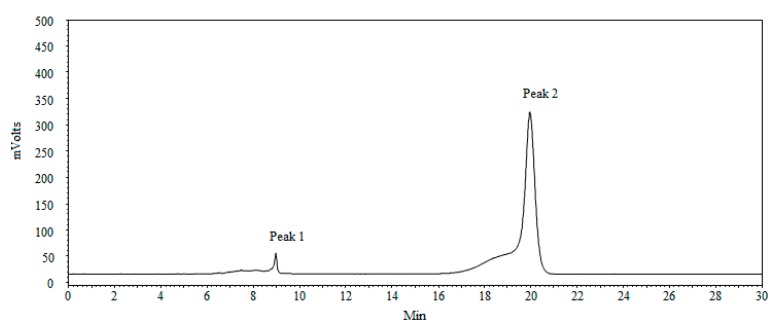
The HPGPC spectrum of PSA.

As shown in Figure 6, the molecular weight distribution of PSA was composed of two main fractions. By employing a calibration curve of Dextran standards (log *M*_W_ = −0.00321*x* + 7.917, *R*^2^ = 0.998), the average molecular weights of these two PSA fractions were calculated to be 5.34 × 10^3^ and 6.27 × 10^5^ Da, respectively.

**Figure 7:**
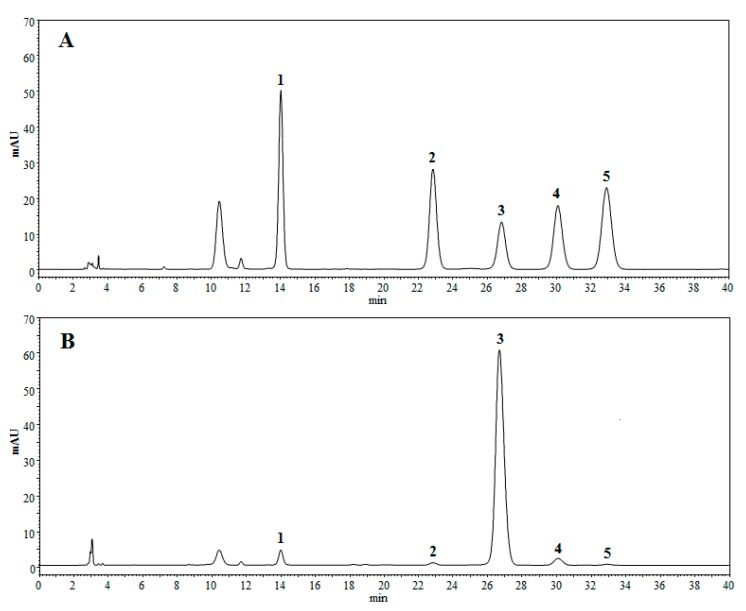
HPLC chromatograms of hydrolyzed polysaccharide derivatives. (**A**) HPLC chromatogram of monosaccharide standards; (**B**) HPLC chromatogram of PSA. (1) mannose, (2) galactose uronic acid, (3) glucose, (4) galactose, (5) arabinose.

Results of the monosaccharide composition of PSA are shown in Figure 7. It was found that PSA consisted of mannose, galactose uronic acid, glucose, galactose, and arabinose with a molar ratio of 1.83:0.55:75.75:1.94:0.45. The monosaccharide compositional analysis implied that glucose is the main monosaccharide in PSA.

### 2.6. Antioxidant Activity of PSA In Vitro

#### 2.6.1. DPPH Radical Scavenging Assay

**Figure 8:**
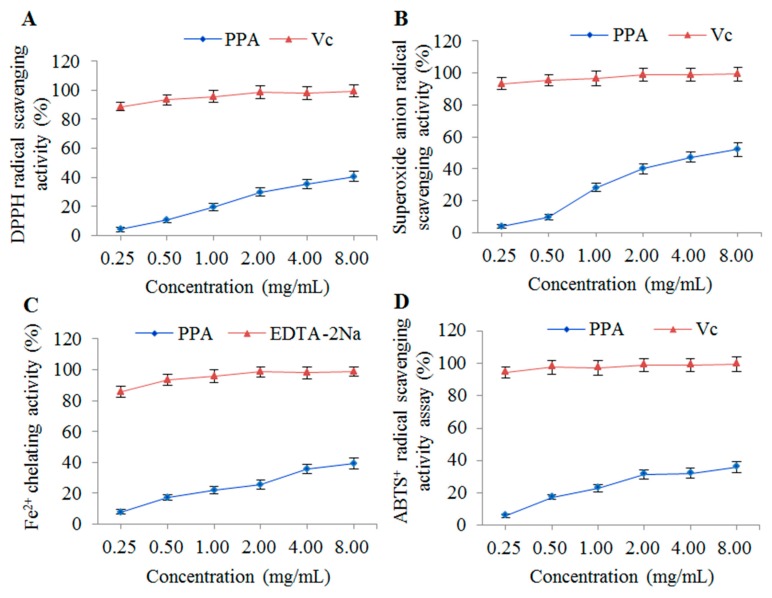
Antioxidant activities of PSA. (**A**) DPPH radical scavenging assay; (**B**) superoxide anion radical scavenging assay; (**C**) Fe^2+^ chelating assay; (**D**) ABTS^+^ radical scavenging assay.

The DPPH free radical scavenging assay is widely used to evaluate the antioxidant activities of polysaccharides. DPPH is a relatively stable free radical which can be reduced by donating hydrogen or electrons to an antioxidant to form a stable diamagnetic molecule, and the color changes from purple to yellow as the absorbance decreases [37]. The results of the DPPH radical scavenging assay are shown in Figure 8A. The DPPH free radical scavenging activity of PSA increased slowly with the increase of concentration. The maximum scavenging rates of PSA and ascorbic acid (Vc) was 40.51% and 99.40% at the concentration of 8 mg/mL, respectively. The equivalent DPPH scavenging activity of PSA was approximately 2.27 mg Vc/g. Thus, the results indicated that PSA possessed significant scavenging activity in DPPH-radicals.

#### 2.6.2. Superoxide Anion Radical Scavenging Assay

The superoxide anion free radical is a relatively weak oxidant produced by various biological and photochemical reactions. It can react with numerous biomolecules to form a stronger reactive oxygen species (ROS) and induce damage to lipids, proteins and DNA [38,39]. The superoxide radical scavenging activities of PSA were concentration-dependent from 0.25 to 8 mg/mL (Figure 8B). The scavenging rate of PSA reached maximum value of 52.12% at 8 mg/mL and that of Vc was 99.29%. The equivalent superoxide anion radical scavenging activity of PSA was approximately 1.26 mg Vc/g. Therefore, PSA exhibited a moderate scavenging effects on superoxide anion radicals.

#### 2.6.3. Fe^2+^ Chelating Assay

Iron-chelating can produce antioxidant effects due to the formation of cross bridges between the carboxyl group in uronic acid and divalent ions. The complex formation can be disrupted by chelating agents and the red color decreases [24,40]. The results of Fe^2+^ chelating assay were shown in Figure 8C. The Fe^2+^ chelating activity of PSA was concentration-dependent at the concentrations of 0.25–8 mg/mL. However, PSA showed much lower chelating activity compared with EDTA-2Na with chelating activities of 39.44% at the concentration of 8 mg/mL. The equivalent Fe^2+^ chelating activity of PSA was approximately 0.24 mg EDTA-2Na/g. Therefore, PSA has potential in preventing oxidative damage due to the chelating effects of ferrous ions.

#### 2.6.4. ABTS^+^ Radical Scavenging Assay

The ABTS^+^ radical scavenging assay is often used to evaluate the total antioxidant potential of chemical components. ABTS^+^ is a peroxidase substrate that can be oxidized by oxidants to produce a metastable radical cation (the specific absorbance at 734 nm). Antioxidants can donate electrons or hydrogen atoms to the radical cation and result in discoloration [28,41]. As shown in Figure 8D, the ABTS^+^ radical scavenging activity of PSA was concentration-dependent, and reached a maximum scavenging rate (35.86%) at the concentration of 8 mg/mL. The results indicated that PSA possessed significant ABTS radical scavenging activity, which was lower however than that of Vc. The equivalent ABTS^+^ radical scavenging activity of PSA was approximately 1.21 mg Vc/g.

### 2.7. Antimicrobial Activities of PSA

The antimicrobial activities of PSA were assessed by MIC and MBC tests on *Escherichia coli*, *Staphylococcus aureus*, and *Candida albicans.* The results are shown in Table 3. PSA exerted a moderate activity against the Gram-negative strain *Escherichia coli* and the Gram-positive strain *Staphylococcus aureus*, with MICs of 8 and 16 mg/mL. The MBCs of PSA on these two strains were not obtained in this study indicating that antimicrobial action of PSA was bacteriostatic. In addition, no significant effect of PSA was found against *Candida albicans* under the tested concentrations. The results indicated that PSA has potentials to be explored as biological bacteriostatic agent in food or medicine industry [42].

## 3. Materials and Methods

### 3.1. Materials and Chemicals

PRPCA were purchased from Sichuan Neautus Traditional Chinese Medicine Co. Ltd. (Chengdu, China). A specimen was stored at College of Pharmacy, Chengdu Traditional Chinese Medicine (Chengdu, China). DPPH (1,1-diphenyl-2-picrylhydrazyl), EDTA-2Na, ascorbic acid, and ABTS^+^ and dextrans with different molecular weights were purchased from Sigma Chemicals Co. (St. Louis, MO, USA). The monosaccharide standards of mannose, glucose, galactose, galactose acid, and arabinose were purchased from National Institute for Food and Drug Control (Beijing, China). 3-Methyl-1-phenyl-2-pyrazolin-5-one (PMP) was obtained from the SinoPharm Chemical Reagents Co., Ltd. (Shanghai, China). All other chemicals and reagents used in the experiments were of analytical grade.

### 3.2. Extraction of PSA by Ultrasonic-Assisted Extraction (UAE)

Preserving the structure of polysaccharides during extraction and isolation is crucial to maintain their bioactivity. Therefore, conventional methods for polysaccharide extraction, such as heating water extraction, have been gradually abandoned due to hydrolysis, ionization, or oxidation as a result of longer extraction time [43]. UAE has been developed to improve extraction by reducing energy and time while obtaining higher bioactivity, and it has been widely used to extract polysaccharides from natural products [19,20].

In this study, UAE was employed for the extraction of PSA. The dried PRPCA were powdered and 2.0 g sample was extracted by distilled water on an ultrasonic cleaning machine (Tianjin Autoscience Instrument Co., Ltd, Tianjin, China). The following extraction conditions were used: ultrasonic power (140–300 W), extraction time (20–60 min), ultrasonic temperature (40–80 °C) and ratio of water to raw material (10–50 mL/g). After extraction, the solutions were collected and centrifuged (4000 rpm, 10 min), and the supernatants were concentrated to an appropriate volume under reduced pressure at 50 °C using a RE-52AA rotary evaporator (Yarong Biochemical Instrument, Shanghai, China). Three volumes of anhydrous alcohol were added to precipitate polysaccharides overnight at 4 °C [15]. The crude polysaccharides were then obtained after centrifugation at 5000 rpm for 10 min. After being washed with anhydrous ethanol, acetone and diethyl ether, the polysaccharides were air-dried at 50 °C. The contents of the polysaccharides were measured by the phenol-sulfuric method as previously reported [44]. Glucose was used as standard and the yield (%) of polysaccharides (PSA) was calculated using Equation (2):

Yield (%) = *W*_1_/*W*_0_ (2)

where *W*_1_ was the content of crude polysaccharides (g), and *W*_0_ was weight of dried PRPCA (g).

### 3.3. Experimental Design

Classical methods to optimize the process variables (changing one variable at a time while keeping the others at fixed levels) are laborious and time-consuming that often cannot guarantee the determination of optimal conditions [45]. Carrying out experiments with every possible combination of all the variables is impractical because quite a large number of experiments are required [46]. Therefore, a RSM design was applied to construct a second-order polynomial model to investigate the best possible experimental conditions that maximize the PSA extraction yield. However, preliminary tests are needed to simplify the variables and factors before the RSM was carried out [15]. Thus, preliminary experiments of PSA extraction were performed in this study.

On the basis of the preliminary single-factor experiments, a three-factor Box–Behnken design (BBD) was employed. Three independent variables including extraction time (*X*_1_, 30, 40 and 50 min), ultrasonic temperature (*X*_2_, 60, 70 and 80 °C) and ultrasonic power (*X*_3_, 180, 220 and 260 W) were investigated and the three levels were coded −1, 0 and +1, respectively (Table 4). The yield of obtained polysaccharides was calculated as the response dependent value. The following second-order polynomial Equation (3) was used to perform the relationship between factors and the response [47]:

(3) $$Y=\beta_{0}+\sum_{i=1}^{3}\beta_{i}X_{i}+\sum_{i=1}^{3}\beta_{ii}X_{i}^{2}+\sum_{i=1}^{3}\sum_{j=i+1}^{3}\beta_{ij}X_{i}\beta_{i}X_{j}$$

where *Y* is the predicted response; *β*_0_ is the constant of the model, *β_i_* is the linear coefficient of the model, *β_ii_* is the second-order interaction of the model, and *β_ij_* is quadratic coefficient of the model. *X_i_* and *X_j_* are the independent variables.

### 3.4. Ultraviolet (UV) and Infrared Spectroscopy (IR) Analysis

UV analysis is often used to detect whether proteins or nucleic acids are present in polysaccharides [21] and Fourier transform infrared spectroscopy (FT-IR) analysis is performed to identify the fundamental groups present in polysaccharides’ structures [48]. Therefore, UV and FT-IR analysis were carried out in this study. A certain concentration of PSA was prepared using deionized water, and ultraviolet spectrum scan was performed on a TU-1901 ultraviolet visible spectrophotometer (Beijing Purkinje General Instrument Co., Ltd., Beijing China) in the range of 200–400 nm. The FT-IR analyses of PSA were carried out using the KBr-disk method as previously reported [49]. The PSA powder and KBr powder were thoroughly mixed and pressed into a 1-mm pellet. The spectrum was determined using a TENSOR 37 FT-IR spectrophotometer (Bruker, Ettlingen, Germany) between the frequency range of 500–4000 cm^−1^.

### 3.5. Scanning Electron Microscopy (SEM)

Scanning electron microscopy (SEM) can provide visual evidence of the shape and surface characteristics of polysaccharides [9]. In this study, the PSAs were examined with a scanning electron microscope system (JSM-7001F, JEOL, Tokyo, Japan). The samples were glued on specimen stubs by coating with a thin layer of platinum under high vacuum conditions at 8.0 kV acceleration voltage. In addition, images were obtained under different magnifications of 10,000×, 5000×, 2000× and 1000×, respectively.

### 3.6. Molecular Weight Determination

It has been reported that the molecular weight affects the bioactivities of polysaccharides [50]. The molecular weights of PSA was determined by high performance gel permeation chromatography (HPGPC) on an Agilent 1260 HPLC system (Agilent, Santa Clara, CA, USA) equipped with an evaporative light scattering detector (ELSD). The sample separation was performed on a TSK-Gel G4000 SWXL (7.8 mm × 30 cm, 8 μm, Tosoh Corp, Tokyo, Japan) column. PSA was dissolved in deionized water (5 mg/mL) and eluted with deionized water at a flow rate of 0.6 mL/min with column temperature of 35 °C. The injection volume was 20 μL. A series of dextran standards were used to calibrate the linear regression.

### 3.7. Monosaccharide Composition Analysis of PSA

It is widely recognized that the monosaccharide composition is an important factor closely related to the bioactivities of natural polysaccharides [51]. Monosaccharide composition of PSA was determined by HPLC as previously described with some modifications [52]. PSA (10 mg) was hydrolyzed by trifluoroacetic acid (TFA, 2 mol/L) at 110 °C for 5 h. Then TFA was removed under reduced pressure, and the residue was washed by methanol for three times before re-dissolving in 2 mL deionized water. Subsequently, 1-phenyl-3-methyl-5-pyrazolone (PMP) methanol solution (0.5 mol/L, 0.2 mL) and NaOH solution (0.3 mol/L, 0.2 mL) were added to the hydrolysate and incubated at 70 °C for 1 h. The mixture was neutralized by adding HCl solution (0.3 mol/L, 0.2 mL). Finally, trichloromethane (1 mL) was added and extracted for three times. The aqueous phase was collected and diluted to 5mL by deionized water. The sample (10 μL) was analyzed by an Agilent 1260 HPLC system equipped with an UV detector set at 245 nm. The separation was performed on a Phenomenex Gemini 5μ C18 110A column (4.6 mm × 250 mm, 5 μm) at a flow rate of 0.8 mL/min at 35 °C. The mobile phase was a mixture of phosphate buffer (0.05 mol/L, PH = 6.8) and acetonitrile (82:18, *v*/*v*).

### 3.8. In Vitro Antioxidant Activities

Natural materials, especially polysaccharides, are a highly promising source of antioxidants [51]. However, it’s reported that various types of antioxidant capacity measurements are required in order to take into account the different mechanisms of antioxidant action [53]. Thus, DPPH radical scavenging, superoxide anion radical scavenging, Fe^2+^ chelating and ABTS^+^ radical scavenging assays were performed in this study.

#### 3.8.1. DPPH Radical Scavenging Activity

The DPPH radical scavenging activity assay of PSA was performed by the method according to a previous report with some modifications [24]. Sample solution (2 mL, prepared in the deionized water) was added into 2 mL DPPH (1 × 10^−4^ mol/L in ethanol) and kept in dark for 30 min after shaking with vortex at room temperature. After centrifugation at 5000 rpm for 10 min, the absorbance was measured at 517 nm by ultraviolet visible spectrophotometry. Vc was used as a control at the same concentration. The DPPH free radical scavenging activity of PSA was calculated using the following Equation (4):

(4) $$\mathrm{DPPH}\;\mathrm{scavenging}\;\mathrm{activity}\;\left(\%\right)=\left(1-\frac{A_{2}-A_{1}}{A_{0}}\right)\times 100\%$$

where *A*_0_ is the absorbance of methanol plus DPPH-methanol solution. *A*_1_ is the absorbance of methanol plus samples with different concentrations. *A*_2_ is the absorbance of DPPH-methanol solution plus samples with different concentrations.

#### 3.8.2. Superoxide Anion Radical Scavenging Activity

The superoxide anion radical scavenging activity assay was carried out based on the previous report with some modifications [10]. Briefly, Tris-HCl buffer (4.5 mL, 50 mM, pH 8.2) was incubated at 25 °C for 20 min, and mixed with pyrogallic acid (0.5 mL, 25 mM) and sample solution (1 mL). The mixture was then incubated at 25 °C for 5 min. Subsequently, 8 mM HCl (1 mL) was added to the mixture to terminate the reaction, and the absorbance was measured at 320 nm. The Vc was used as a positive control. The ability to scavenge superoxide radicals was calculated as Equation (5):

(5) $$\mathrm{Superoxide}\;\mathrm{scavenging}\;\mathrm{activity}\;\left(\text{\%}\right)=\left(1-\frac{A_{2}-A_{1}}{A_{0}}\right)\times 100\%$$

where *A*_0_ is the absorbance of negative control (deionized water instead of sample solution). *A*_1_ is the absorbance of the sample background (without pyrogallic acid). *A*_2_ is the absorbance of the tested sample.

#### 3.8.3. Fe^2+^ Chelating Activity

The Fe^2+^ chelating activity of PSA was determined according to a previous report with minor modifications [54]. Briefly, sample solution (1 mL) was added to deionized water (3.7 mL), ferrous chloride solution (0.1 mL, 2 mM) and ferrozine solution (0.2 mL, 5 mM). The mixture was then incubated at 25 °C for 10 min, and the absorbance was determined at 562 nm. Ethylenediamine tetraacetic acid disodium salt (EDTA-2Na) was used as the positive control. The following Equation (6) was used to calculate the Fe^2+^ chelating activity:

(6) $${\mathrm{Fe}}^{2+}\;\mathrm{chelating}\;\mathrm{activity}\;\left(\%\right)=\left(1-\frac{A_{2}-A_{1}}{A_{0}}\right)\times 100\%$$

where *A*_0_ is the absorbance of negative control (deionized water instead of sample solution). *A*_1_ is the absorbance of the sample background (without ferrous chloride solution). *A*_2_ is the absorbance of the tested sample.

#### 3.8.4. ABTS^+^ Radical Scavenging Activity

The ABTS^+^ radical scavenging activity was assayed by a previously described method with some modifications [55]. Potassium persulphate solution (2.5 mM) and ABTS^+^ solution (7 mM) were mixed and kept in the dark for 12–16 h at room temperature. The prepared ABTS^+^ solution was diluted to the absorbance of 0.70 ± 0.02 (734 nm). Sample solution (0.5 mL) was mixed with 3.5 mL of the diluted ABTS^+^ solution, and reacted for 6 min at room temperature. The absorbance was then measured at 734 nm and the Vc was used as a positive control. The ABTS radical scavenging activity was calculated according to Equation (7).

(7) $${\mathrm{ABTS}}^{+}\;\mathrm{scavenging}\;\mathrm{activity}\;\left(\%\right)=\left(1-\frac{A_{2}-A_{1}}{A_{0}}\right)\times 100\%$$

where *A*_0_ is the absorbance of negative control (deionized water instead of sample solution). *A*_1_ is the absorbance of the sample background (without ABTS^+^ solution). *A*_2_ is the absorbance of the tested sample.

### 3.9. Antimicrobial Activities

It has become very important to seek novel antibacterial agents or alternative therapies because resistant strains are not sensitive to the antibiotics and these infections are usually very difficult to treat [56]. Recently, polysaccharides have received increasing attention due to their antimicrobial activities [57]. The antimicrobial activities of PSA were evaluated by minimal inhibition concentration (MIC) and minimal bactericide concentration (MBC) assays based on a previous study with some modifications [58,59]. The Gram-negative strain *Escherichia coli* (ATCC 25922), Gram-positive strain *Staphylococcus aureus* (ATCC 6538), and pathogenic fungus *Candida albicans* (ATCC 10231) were used in this study. Briefly, different concentrations of PSA solution were prepared by serial dilutions. Each well of a 96-well plate received 140 μL of culture medium and 50 μL of PSA solution (final concentrations of 32, 16, 8, 4, 2, 1, 0.5, 0.25 mg/mL). Then 10 μL of each microbial suspension (1 × 10^6^ CFU/mL) were added to the wells. After inoculation, the plate was incubated at 37 °C for 24 h. The MIC was determined as the lowest concentration that completely suppressed the growth of the microorganisms. The MBC was determined by sub-culturing the test dilutions without microorganism growth on agar plates. After incubating for 24 h, the MBC was determined as the concentration at which no growth was observed on subculture.

### 3.10. Statistical Analysis

All the experiments were carried out in triplicate and the data are presented as means ± S.D. Analysis of the Box-Behnken design and data was carried out using Analysis of variance (ANOVA) by Design Expert software version 8.0.6.1 (Stat-Ease Inc., Minneapolis, MN, USA). A value of *p* < 0.05 was regarded as statistically significant.

## 4. Conclusions

The extraction conditions of PSA by UAE method were optimized by RSM as follows: ratio of water to raw material of 30 mL/g, extraction time of 46.50 min, ultrasonic temperature of 72.00 °C, and ultrasonic power of 230 W. Under these conditions, the obtained PSA yield was 13.21 ± 0.37%, which closely agreed with the value predicted by the model. The PSA was characterized by FT-IR and proved to contain no polypeptides or proteins by UV detection. SEM analysis showed the surface of PSA appeared to be relatively smooth, with some honeycomb cavities and fine stripes. The average molecular weights of the PSA were estimated to be 5.34 × 10^3^ and 6.27 × 10^5^ Da, respectively. The monosaccharide composition of PSA consisted of mannose, galactose uronic acid, glucose, galactose, arabinose, and of which glucose is the main monosaccharide. Furthermore, PSA exhibited considerable concentration-dependent antioxidant activity in vitro. PSA also exerted a moderate activity against *Escherichia coli* and *Staphylococcus aureus*, with MICs of 8 and 16 mg/mL, respectively. Collectively, this study provides a promising strategy to obtain bioactive polysaccharides from processed products of herbal medicines. However, more investigations are needed to purify the polysaccharides and reveal the relationship between the structure and the bioactivities.

## Figures and Tables

**Table 1:** Box–Behnken experimental design and results for extraction yields.

Run	Extraction Time (*X*_1_) (min)	Ultrasonic Temperature (*X*_2_) (°C)	Ultrasonic Power (*X*_3_) (W)	Extraction Yield (%)	
				Actual Value	Predicted Value
1	50.00	80.00	220.00	11.93	11.80
2	50.00	70.00	180.00	11.26	11.48
3	40.00	70.00	220.00	12.77	12.61
4	40.00	80.00	180.00	9.44	9.34
5	40.00	60.00	260.00	9.03	9.13
6	40.00	70.00	220.00	12.59	12.61
7	40.00	70.00	220.00	12.88	12.61
8	40.00	80.00	260.00	11.82	11.81
9	30.00	70.00	260.00	10.91	10.69
10	50.00	70.00	260.00	11.96	12.09
11	50.00	60.00	220.00	11.14	10.91
12	30.00	60.00	220.00	6.13	6.25
13	30.00	80.00	220.00	10.09	10.32
14	30.00	70.00	180.00	6.89	6.76
15	40.00	70.00	220.00	12.36	12.61
16	40.00	70.00	220.00	12.43	12.61
17	40.00	60.00	180.00	7.05	7.06

**Table 2:** ANOVA for the response surface quadratic model.

Source	Sum of Squares	df	Mean Square	*F* Value	*p*-Value (Prob > *F*)
Model	76.16	9	8.46	121.22	<0.0001
*X*_1_-Extraction time	18.82	1	18.82	269.59	<0.0001
*X*_2_-Ultrasonic temperature	12.33	1	12.33	176.57	<0.0001
*X*_3_-Ultrasonic power	10.31	1	10.31	147.63	<0.0001
*X*_1_*X*_2_	2.51	1	2.51	35.99	0.0005
*X*_1_*X*_3_	2.76	1	2.76	39.47	0.0004
*X*_2_*X*_3_	0.040	1	0.040	0.57	0.4738
*X*_1_^2	3.66	1	3.66	52.36	0.0002
*X*_2_^2	14.44	1	14.44	206.83	<0.0001
*X*_3_^2	8.48	1	8.48	121.50	<0.0001
Residual	0.49	7	0.070		
Lack of Fit	0.29	3	0.098	2.03	0.2523
Pure Error	0.19	4	0.048		
Cor Total	76.65	16			
Standard deviation	0.26	*R*^2^	0.9936		
C.V.%	2.09	Adj *R*^2^	0.9854		
Adeq Precision	31.341	Pred *R*^2^	0.9345		

**Table 3:** Antibacterial and antifungal activities of PSA.

Microorganism	PSA (mg/mL)	
	MIC	MBC
*Escherichia coli*	8	>32
*Staphylococcus aureus*	16	>32
*Candida albicans*	>32	>32

**Table 4:** Independent variables and their levels used for Box-Behnken design (BBD).

Independent Variables	Coded Levels of Variables		
	−1	0	1
Extraction time (*X*_1_) (min)	30	40	50
Ultrasonic temperature (*X*_2_) (°C)	60	70	80
Ultrasonic power (*X*_3_) (W)	180	220	260
